# NMR-based solution structure of the *Caulobacter crescentus* ProXp-ala *trans*-editing enzyme

**DOI:** 10.1007/s12104-024-10193-3

**Published:** 2024-08-31

**Authors:** Antonia D. Duran, Eric M. Danhart, Xiao Ma, Alexandra B. Kuzmishin Nagy, Karin Musier-Forsyth, Mark P. Foster

**Affiliations:** 1 Center for RNA Biology, Department of Chemistry and Biochemistry, The Ohio State University, Columbus, OH 43210 USA; 2 484 W 12th Ave, Columbus, OH 43017 USA

**Keywords:** ProXp-ala, tRNA-editing, *Trans*-editing, NMR structure

## Abstract

ProXp-ala is a key component of the translational machinery in all three Domains of life. This enzyme helps to maintain the fidelity of proline codon translation through aminoacyl-tRNA^Pro^ proofreading. In the first step of tRNA aminoacylation, the cognate aminoacyl-tRNA synthetase (aaRS) binds and activates an amino acid in the enzyme’s synthetic active site. If a non-cognate amino acid passes this first selection step and is charged onto the tRNA, a distinct aaRS editing active site may recognize the mischarged tRNA and deacylate it. Alternatively, this editing reaction may be carried out by a separate enzyme that deacylates the mischarged tRNA *in trans*. ProXp-ala is responsible for editing Ala mischarged onto tRNA^Pro^. Since *trans*-editing domains such as ProXp-ala bind their substrates after release from the synthetase, they must recognize not only the mischarged amino acid, but also the specific tRNA. Previous studies showed that *Caulobacter crescentus* (*Cc*) ProXp-ala distinguishes tRNA^Pro^ from tRNA^Ala^, in part, based on the unique tRNA^Pro^ acceptor stem base pair C1:G72. Previous crystallographic and NMR data also revealed a role for conformational selection by the ProXp-ala α2 helix in Ala- versus Pro-tRNA^Pro^ substrate discrimination. The α2 helix makes lattice contacts in the crystal, which left some uncertainty as to its position in solution. We report resonance assignments for the substrate-free *Cc* ProXp-ala and the NMR-derived three-dimensional structure of the protein. These data reveal the position of the α2 helix in solution, with implications for substrate binding and recognition.

**Supplementary Information:**

The online version contains supplementary material available at 10.1007/s12104-024-10193-3.

## Biological context

Protein translation fidelity is fundamental in the central dogma of biology. Alterations in amino acid sequence of a protein can cause changes in structure, stability and function, leading to disease states or cell death (Zaher and Green 2009). Aminoacyl tRNA synthetases play a key role in this process by first activating the cognate amino acid with ATP to form an aminoacyl adenylate intermediate (Ibba and Söll 2000), then transfer the activated amino acid onto the 3’ acceptor end of the corresponding tRNA. Mistakes in charging a tRNA with the wrong amino acid will result in miscoding if the errant amino acid is not removed prior to its recruitment to ribosomes. In pre-transfer editing, the noncognate aminoacyl-adenylate intermediate is hydrolyzed and is not charged onto the tRNA (Jakubowski and Goldman 1992). However, activated noncognate amino acids are sometimes mischarged onto the tRNA, requiring post-transfer editing to correct (Jakubowski and Goldman 1992).

Several strategies have been described that help to avoid events that lead to proline codon mistranslation, as mutations to and from proline in particular can cause deleterious changes in protein structure due to proline’s uniquely constrained phi angle (Morgan and Rubenstein 2013).

Size and chemical selection prevent most incorrect amino acids from binding in the active site of ProRS, but alanine and cysteine can pass through that filter and be misactivated (Beuning and Musier-Forsyth 2000; Ahel et al. 2002). The majority of bacterial ProRSs possess an insertion (INS) domain that deacylates mischarged Ala-tRNA^Pro^ (*cis* editing) (Wong et al. 2002). Many organisms that express ProRSs lacking the INS editing domain, express a free-standing editing domain known as ProXp-ala that performs a similar function (Vargas-Rodriguez and Musier-Forsyth 2013). Additionally, many bacteria use a triple-sieve mechanism whereby Ala-tRNA^Pro^ is hydrolyzed in *cis* or in *trans*, and Cys-tRNA^Pro^ is deacylated by YbaK, another single-domain *trans*-editing enzyme (An and Musier-Forsyth 2004).

*Trans*-editing enzymes, by definition, recognize the mischarged tRNA after it is released from the synthetase, recognizing either the tRNA acceptor stem elements or the aminoacyl moiety, or both. Previous studies of *Caulobacter crescentus* (*Cc*) ProXp-ala showed that residue Arg80 is important for recognizing the terminal C1:G72 base pair of tRNA^Pro^ (Ma et al. 2023). Attempts to use NMR chemical shift perturbations to map Ala recognition by titration with free Ala or Ala amide failed to detect binding at concentrations up to 9 mM (Ma 2021). ProXp-ala binds a non-hydrolysable Ala-tRNA^Pro^ acceptor stem mimic, nh-microhelix^Pro^, with a 0.52 µM K_d_ (Danhart et al. 2017). Additionally, ACCA-Ala failed to inhibit deacylation of tRNA^Pro^ by ProXp-ala, while nh-microhelix^Pro^ has an inhibitory effect (Ma 2021). Taken together, these observations suggest that the presence of the tRNA terminal base pair is required for recognition of the Ala moiety by ProXp-ala.

Crystal structures of *Cc* ProXp-ala reveal differences in the position of the helix α2, which abuts the presumed active site (Danhart et al. 2017). Both available *Cc* ProXp-ala crystal forms feature lattice contacts at the α2 helix, including hydrophobic packing of phenylalanine residues that could mask the conformation of the substrate-free enzyme (structures 1VJF and 5VXB). Moreover, NMR relaxation experiments show that helix α2 exhibits dynamics on the µs-ms timescale (Danhart et al. 2017), consistent with a propensity for conformational change. These observations suggest that the conformational fluctuations in helix α2 play a role in substrate binding, though the details of this mechanism have not yet been fully described. In this paper, we report backbone and side chain resonance assignments for substrate-free *Cc* ProXp-ala, the NMR-derived three-dimensional structure of the protein, and comment on the position of the α2 helix and implications for substrate binding and recognition.

## Methods and experiments

### Protein production and purification

ProXp-ala was prepared as described previously (Vargas-Rodriguez and Musier-Forsyth 2013). Briefly, the gene encoding *Cc* ProXp-ala with an N-terminal His_6_ metal affinity tag was cloned into pET15b (Novagen) and proteins were expressed in *Escherichia coli* BL21-CodonPlus (DE3)-RIL cells (Agilent) upon induction with 0.1 mM isopropyl β-d-1-thiogalactopyranoside (Gold Biotechnology) for 20 h at room temperature. Uniformly singly-labeled ([U-^15^N]) and doubly-labeled ([U-^13^C, ^15^N]) ProXp-ala samples were prepared in M9 minimal medium supplemented with 100 µM FeCl_3_, 1 µg/mL thiamine HCl, and 1 µg/mL biotin and containing 1 g/L [^15^N]-ammonium chloride (Cambridge Isotopes) as the sole nitrogen source; 3 g/L [^13^C]-glucose (Cambridge Isotopes) was used as the sole carbon source for ^13^C labeling. His_6_-tagged proteins were purified via His-Select Nickel Affinity Gel chromatography (Sigma-Aldrich) using a 5-250 mM imidazole elution gradient. After buffer exchange into NMR buffer (50 mM sodium phosphate pH 7.5, 10 mM NaCl), the His-tag was cleaved using a Thrombin Cleavage Capture Kit (Novagen) and the free His-tag as well as residual uncut His-tagged protein were removed using His-Select Nickel Affinity Gel chromatography. The resulting protein encodes full-length *Cc* ProXp-ala plus three additional Gly-Ser-His N-terminal amino acids. For the final purification step, ProXp-ala was loaded onto a HiLoad 16/600 Superdex-75 size-exclusion chromatography (SEC) column (Cytiva); the column was run in NMR buffer. The Bio-Rad Protein Assay Kit was used to quantify protein concentrations using bovine serum albumin as a standard.

### NMR spectroscopy and structural analysis

For NMR experiments, ProXp-ala samples in NMR buffer were supplemented with 10% D_2_O (vol/vol) as a lock signal and 0.001% 4,4-dimethyl-4-silapentane-1-sulfonic acid (wt/vol) as an internal chemical shift reference. All spectra were recorded at 25 °C using [U-^15^N] or [U-^13^C/^15^N]-ProXp-ala (0.5–0.7 mM) on a Bruker DRX-600 or DRX-800 spectrometer, each equipped with a Bruker Triple Resonance (TXI) cryoprobe. Sequential backbone assignments were obtained from the following triple-resonance NMR spectra: HNCO, HNCA, HNCOCA, HNCACB, and CBCA(CO)NH (Cavanagh 1996). Sidechain assignments were obtained from the following triple-resonance NMR spectra: HCCH-TOCSY, CCCH-TOCSY, and ^15^N-edited-NOESY, ^13^C-edited-NOESY (τ_m_ 200 ms). The ^15^N-edited-NOESY contained corrupted free induction decays (analog to digital converter overflows) due to cryoprobe arcing. Overflow points were removed, and non-uniform sampling reconstruction was applied in NMRPipe (Delaglio et al. 1995).

Inter-proton distance restraints were obtained from 3D ^15^N-edited NOESY and 3D ^13^C-edited NOESY spectra. NOESY cross-peaks were analyzed using NMRViewJ (Johnson 2004). Automated NOESY cross-peak assignments were made using CYANA v. 3.98.15 (Güntert and Buchner 2015). Torsion angle constraints were predicted by TALOS-N (Shen and Bax 2013) based on chemical shift assignments. Structure calculation was performed in CYANA (Güntert and Buchner 2015), and structures were visualized using PYMOL (https://pymol.org/).

Per-residue root-mean-square-deviations (RMSD) between the twenty lowest energy conformers, and between the coordinate average of the NMR ensemble and crystal structure 1VJF were calculated using MOLMOL (Koradi et al. 1996).

### Extent of assignments and data deposition

**Fig. 1:**
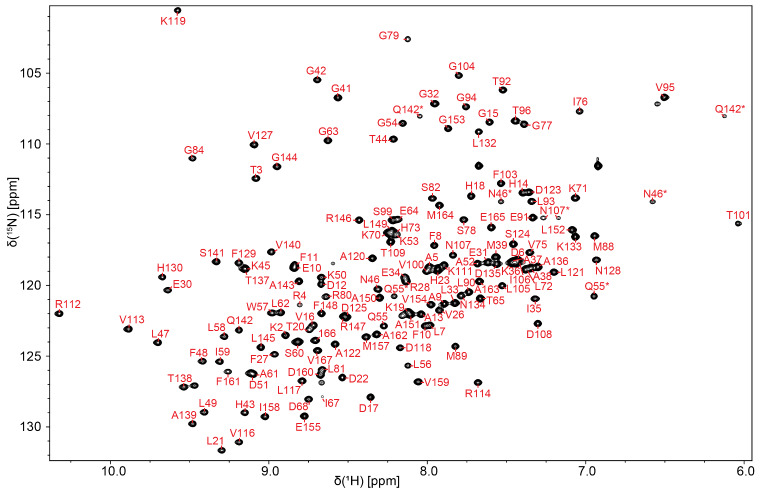
^1^H-^15^N-HSQC spectrum of *Cc* ProXp-ala. Backbone amide signals are labeled by assigned residue. Sidechain signals are indicated by an asterisk (*)

Backbone resonance assignments were 93% (796/855) complete, with 95% (155/163) of ^15^N, 91% (312/342) of ^13^C, and 94% (329/350) of ^1^H resonances assigned, as determined by the worldwide protein databank (wwPDB) validation service. Sidechain assignments were 85% (1067/1263) complete, with 13% (5/39) of ^15^N, 87% (342/394) of ^13^C, and 87% (720/830) of ^1^H resonances assigned. Figure 1 shows a ^1^H-^15^N-HSQC spectrum with signals labeled by residue, representing amide assignments. Resonance assignments and NMR data were deposited to the biological magnetic resonance data bank (BMRB # 52587).

### Secondary structure information

The consensus secondary structure, as determined by CYANA (Schmidt and Güntert 2012), has the topology (α1:R4-A13, β1:K19-L21, α2:L33-A38, β2:K45-K50, β3:L56-A61, α3:L71-K73, β4:S82-F83, α4:Q86-T92, α5:V100-N107, β5:R114-D118, α6(3_10_):L121-D123, β6:T137-A139, α7:Q142-L152, β7:M157-D160, and β8:E165-V166 (Supplementary Fig. 2). This differs from the secondary structures of *Cc* ProXp-ala crystal structures (1VJF and 5VXB), which have an additional β-strand at V127-F129. The absence of β-strand at this position is supported by the chemical shift-based secondary structure prediction (TALOS-N (Shen and Bax 2013), Supplementary Fig. 3), in which only F129 has β-strand propensity, but not the surrounding residues. The lengths of secondary structure elements also differ by 1 to 5 residues compared to the crystal structures.

### NMR structure calculations

**Fig. 2:**
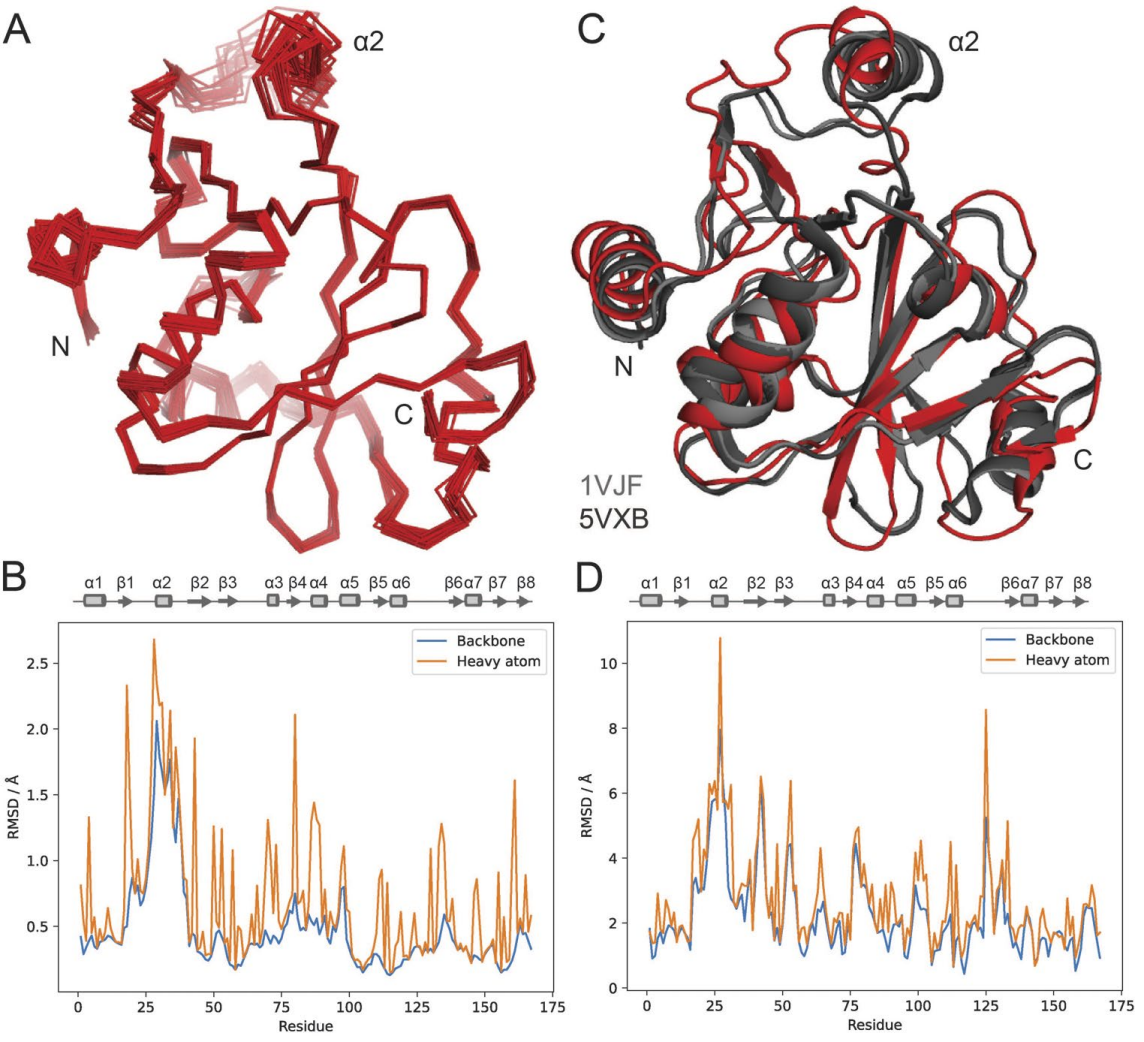
NMR-determined structure of *Cc* ProXp-ala reveals solution-state position of α2 helix. (**A**) Backbone trace of the NMR ensemble of 20 lowest energy structures; amino- and carboxy-terminal ends are labeled, as is helix α2. (**B**) Backbone and heavy atom RMSD between ensemble members. (**C**) Lowest energy model (PDB 9BU5) overlayed with crystal structures of *Cc* ProXp-ala (PDB 1VJF, 5VXB). (**D**) RMSD between ensemble mean (9BU5) and 1VJF

Structure calculation was performed in CYANA v. 3.98.15 (Güntert and Buchner 2015), with crystal structure 1VJF as the starting model (cycle 1 structure) for NOE assignments. The structure calculation included 1986 NOE-based distance restraints (Supplementary Table 1, Supplementary Fig. 4), backbone torsion angle restraints for 159 of the 167 residues, and chi1 torsion angle restraints for 76 of the 167 residues. The resulting ensemble of the 20 lowest energy structures has a backbone RMSD of 0.61 +/- 0.20 Å over the entire protein (residues 1-167), and a heavy atom RMSD of 0.98 +/- 0.16 Å (Fig. 2A, B, Supplementary Fig. 4). The ensemble was deposited to the Protein Data Bank (PDB) under accession code 9BU5. No backbone torsion angles are in the disallowed regions of the Ramachandran plot (Supplementary Fig. 5).

### Position of the α2 helix and residues involved in substrate recognition

**Fig. 3:**
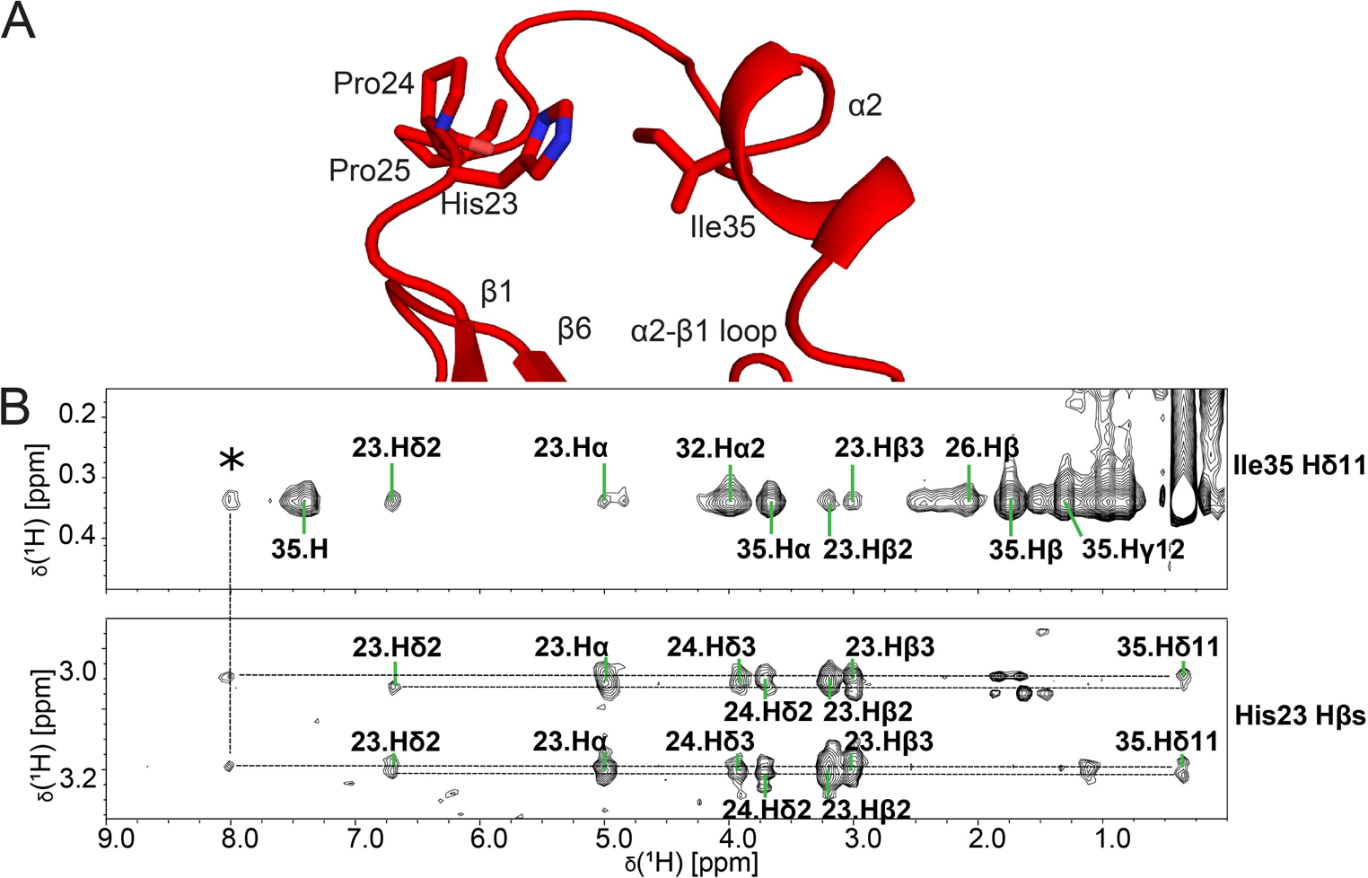
Position of the α2 helix of Cc ProXp-ala is largely defined by NOEs between Ile35 and His23. (**A**) Cartoon depiction of lowest energy model (PDB 9BU5) with Ile35 and His23 sidechain heavy atoms shown as sticks. (**B**) Strip plots from ^13^C-edited NOESY with signals corresponding to Ile35 Hδ1 methyl (top) and His23 Hβs (bottom), with assignments as indicated. Horizontal dashed lines highlight doubling of the His23 Hβ signals, which one set of signals featuring an NOE to a signal at 7.99 ppm (asterisk); this signal is tentatively assigned to His23 Hδ_1_, which is one of the conformations may be protected from exchange

Compared to the crystal structures of *Cc* ProXp-ala, the position of α2 is angled closer to β1 and further away from the substrate binding site (Fig. 2C). As anticipated from the aforementioned crystal contacts and evidence for dynamics in solution, the largest differences in atom coordinates between the ensemble and the available crystal structures are at the α2 helix and preceding loop (Fig. 2D). The position of the α2 helix relative to the preceding strand β1 is defined primarily by covalent structure and a few NOEs between residues Ile35 and His23 (Fig. 3A, B). The His23 Hβ signals in the ^13^C-edited NOESY exhibited doubling (Fig. 3B). One set of these His23 Hβ signals also exhibit an NOE to a proton at 7.99 ppm, which also NOEs to Ile35 Hδ_1_ (Fig. 3B, asterisk). A candidate assignment is His23 Hδ1, which in crystal structures (1VJF and 5VXB) forms a hydrogen bond to the carbonyl oxygen of Pro24. The Pro24 Cβ and Cγ chemical shifts are within the expected range for the *trans* isomer (30.26 ppm for Cβ and 28.07 for Cγ), as seen in crystals. However, because the spectral pattern implies two distinct conformations in this region, those NOEs were excluded for the present structure analysis. The peptide bond Asp125-Pro126 was found to be in the *cis* conformation, with Pro126 chemical shifts of 35.04 ppm for Cβ and 24.66 ppm for Cγ, consistent with the *Cc* ProXp-ala crystal structures.

**Fig. 4:**
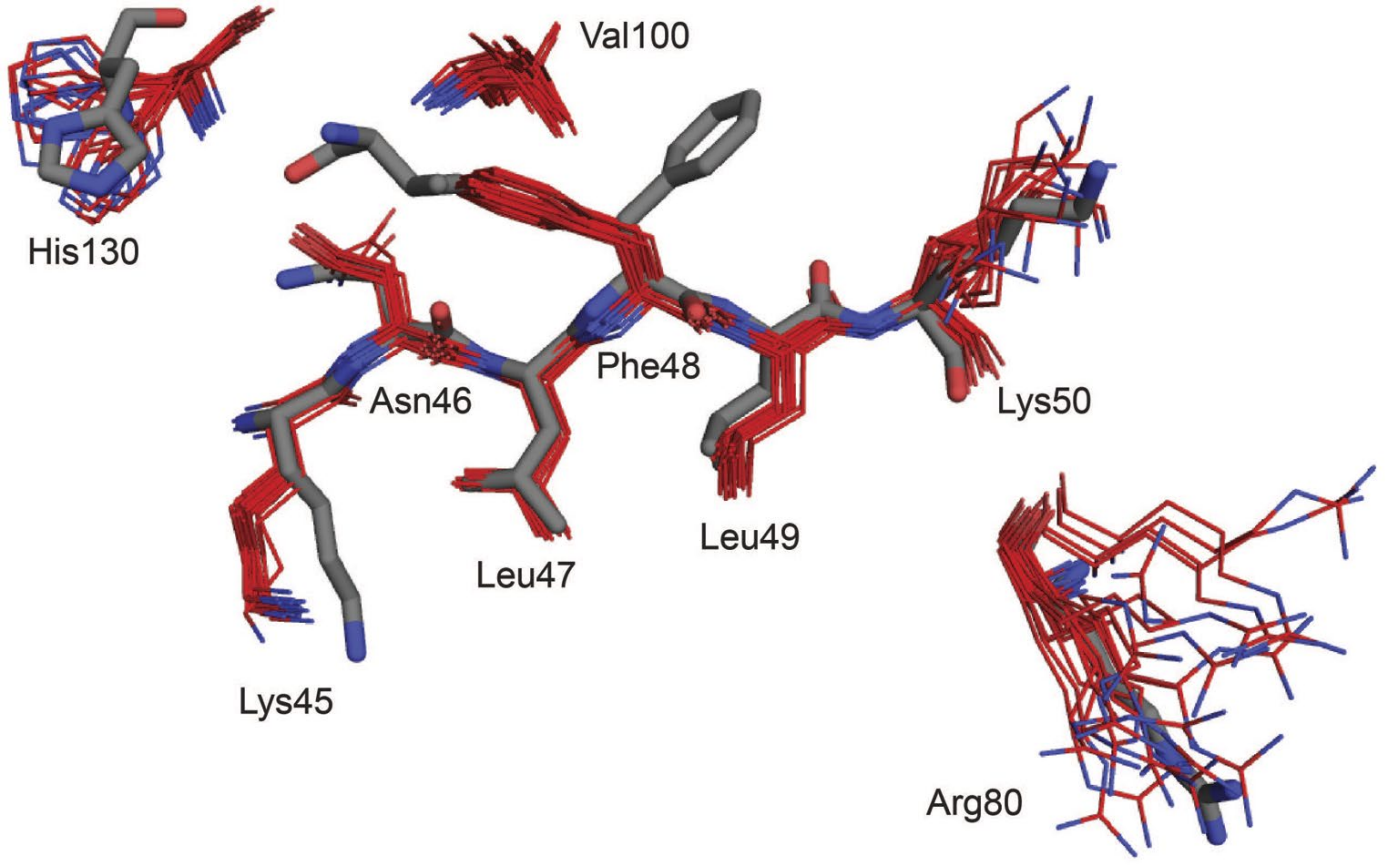
Positions of *Cc* ProXp-ala substrate-binding residues. Heavy atoms of selected residues with known or predicted substrate interactions. NMR ensemble is depicted in red (PDB 9BU5). Crystal structure 1vjf is depicted in grey

Compared to a molecular mechanics energy minimized (MM) docking model of ProXp-ala and Ala-microhelix^Pro^ (Danhart et al. 2017), the α2 helix is in a more open position (away from the substrate binding site). Residues predicted to contact the Ala moiety are also in a more open position compared to the crystal structures (Fig. 4). His130 and Val100 are further from the presumed Ala binding site, though the position of the His130 sidechain is not fully determined in our ensemble. In the MM model, the carbonyl group of Val100 is predicted to hydrogen bond with the amine of the Ala moiety. In our ensemble, Val100 is further away, and the carbonyl is facing away from the presumed binding site. This carbonyl position is supported by the chi1 angle propensity predicted from the assigned chemical shifts. Displacement of Val100 from the presumed binding site may arise from steric clashes with the sidechain of Phe48, which in the ensemble faces towards the binding site, and in the crystal structures faces away. The observed Phe48 chi1 torsion angle is also supported by chemical shift data. Other residues predicted to contact the Ala moiety include Asn46 and Leu47, which are both in similar positions compared to the crystal structures.

Residues shown to contribute to specific *Cc* ProXp-ala binding to the acceptor stem of tRNA^Pro^ include His130, Arg80, and Lys45. His130 prevents Pro-tRNA^Pro^ deacylation (Vargas-Rodriguez et al. 2020). As described above, His130 is further from the binding site, but the position of the sidechain is not precisely defined in the ensemble (Fig. 4). The position of Arg80, which recognizes the tRNA^Pro^ C1:G72 base pair (Ma et al. 2023), is also not well defined. Lys45, which is responsible for positioning the tRNA A76 into the active site (Ma et al. 2023), is in a relatively less extended position and is angled differently compared to the crystal structures.

The open conformation of the α2 helix and substrate binding site observed in the solution NMR structure support a binding model in which *Cc* ProXp-ala forms initial contacts with Ala-tRNA^Pro^, inducing conformational change in which the dynamic α2 helix closes over the Ala moiety. The solution structure of apo *Cc* ProXp-ala sets the stage for structure determination of a complex with its mischarged substrate, Ala-tRNA^Pro^.

## Electronic supplementary material

Below is the link to the electronic supplementary material.

Supplementary Material 1

## Data Availability

Resonance assignments and NMR data were deposited to the biological magnetic resonance data bank (BMRB) under accession code 27185. Structure coordinates were deposited to the protein data bank (PDB) under accession code 9BU5.
