# Supplementary material for: NMR-based solution structure of the Caulobacter crescentus ProXp-ala trans-editing enzyme
Source: Biomol NMR Assign. 2024 Aug 31;18(2):233–8. doi: 10.1007/s12104-024-10193-3 (PMC11511748; doi:10.1007/s12104-024-10193-3)
Supplement: Supplementary file 1 — Supplementary Material 1 [file 12104_2024_10193_MOESM1_ESM.docx]

**Supplementary Figures**


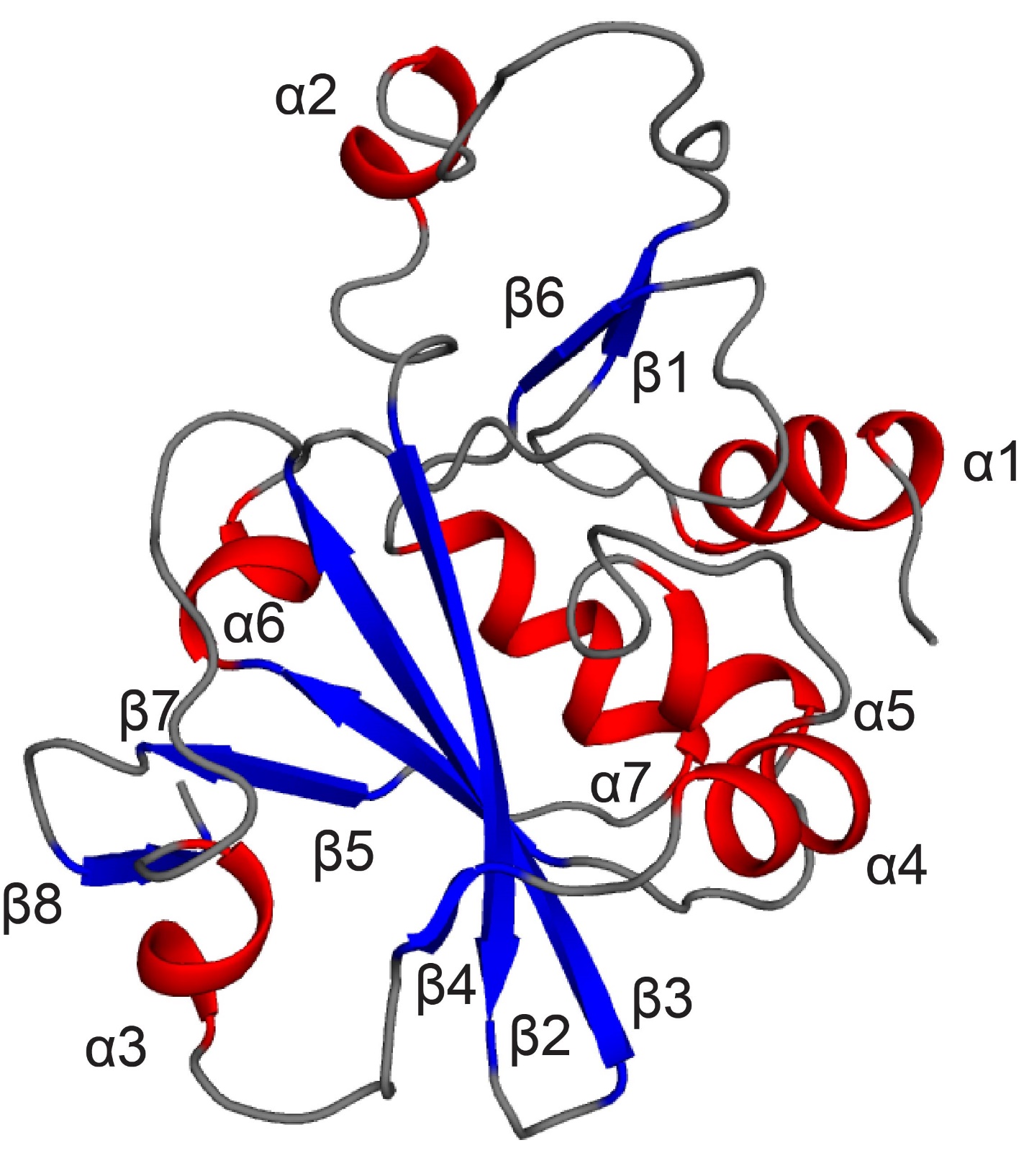


**Supplementary Fig 1:** Secondary structure of *Cc* ProXp-ala, depicted as a cartoon of the lowest energy ensemble member.


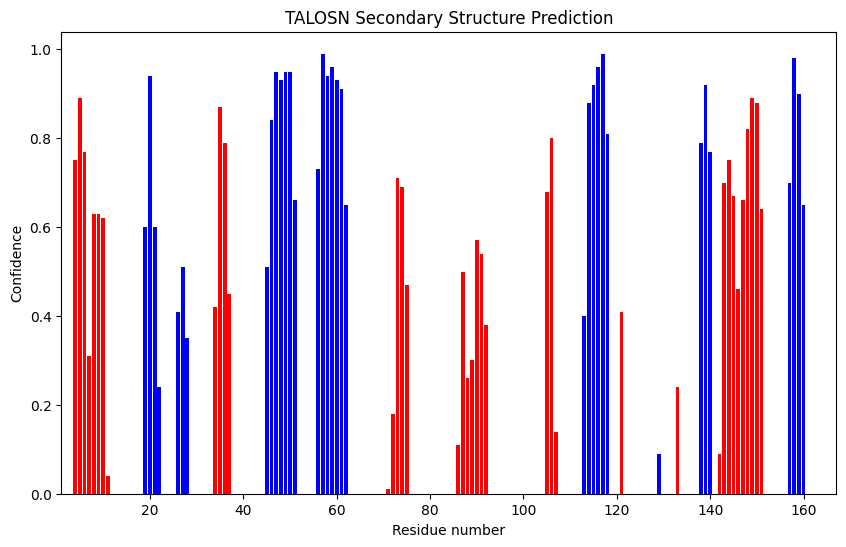


**Supplementary Fig. 2**: TALOS-N secondary structure predictions of *Cc* ProXp-ala. Red bars indicate α-helices. Blue bars indicate β-strands. No bar indicates loop, except for the first and last residues (M1, V167), which do not have secondary structure predictions.

| **Description** | **Value** |
| --- | --- |
| Total distance restraints | 1986 |
| Intra residue (\|i-j\| = 0) | 531 |
| Sequential (\|i-j\| = 1) | 544 |
| Medium range (\|i-j\| > 1 and \|i-j\| < 5) | 360 |
| Long range (\|i-j\| ≥ 5) | 551 |
| Inter-chain | 0 |
| Hydrogen bond restraints | 0 |
| Disulfide bond restraints | 0 |
| Total dihedral-angle restraints | 0 |
| Number of unmapped restraints | 0 |
| Number of restraints per residue | 11.6 |
| Number of long range restraints per residue | 3.2 |

**Supplementary Table 1:** Summary of experimentally observed NMR restraints in *Cc* ProXp-ala structure calculation. Table derived from wwPDB validation report.


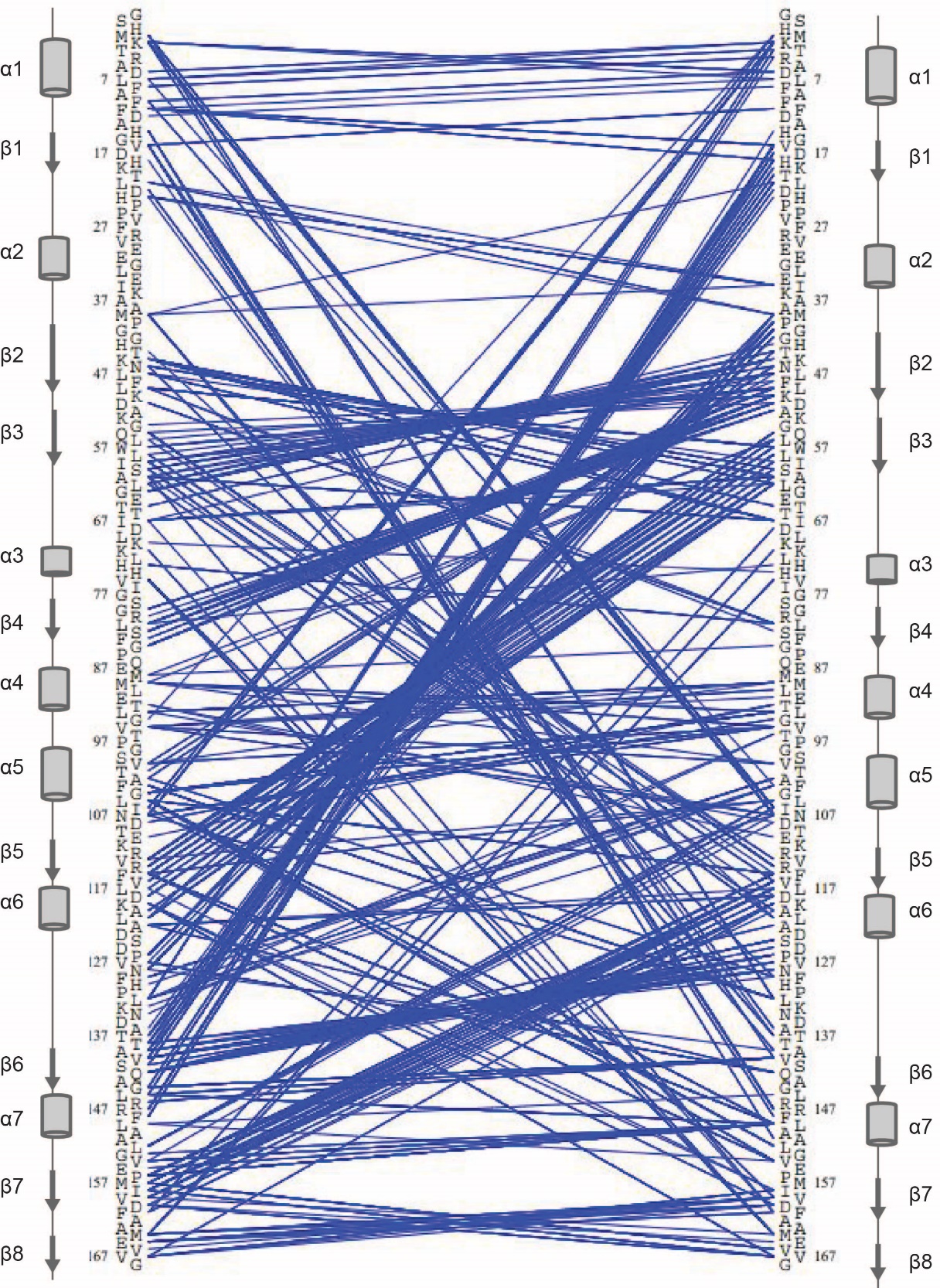


**Supplementary Fig. 3:** Long-range distance restraints of *Cc* ProXp-ala. Restraints five or more residues apart are shown as blue lines connecting identical copies of the protein sequence. Lines from the upper left to lower right represent restraints between side-chain atoms. Lines from the lower left to upper right represent restraints that involve sidechain atoms. Plot generated by CYANA (Schmidt & Güntert, 2012).

­


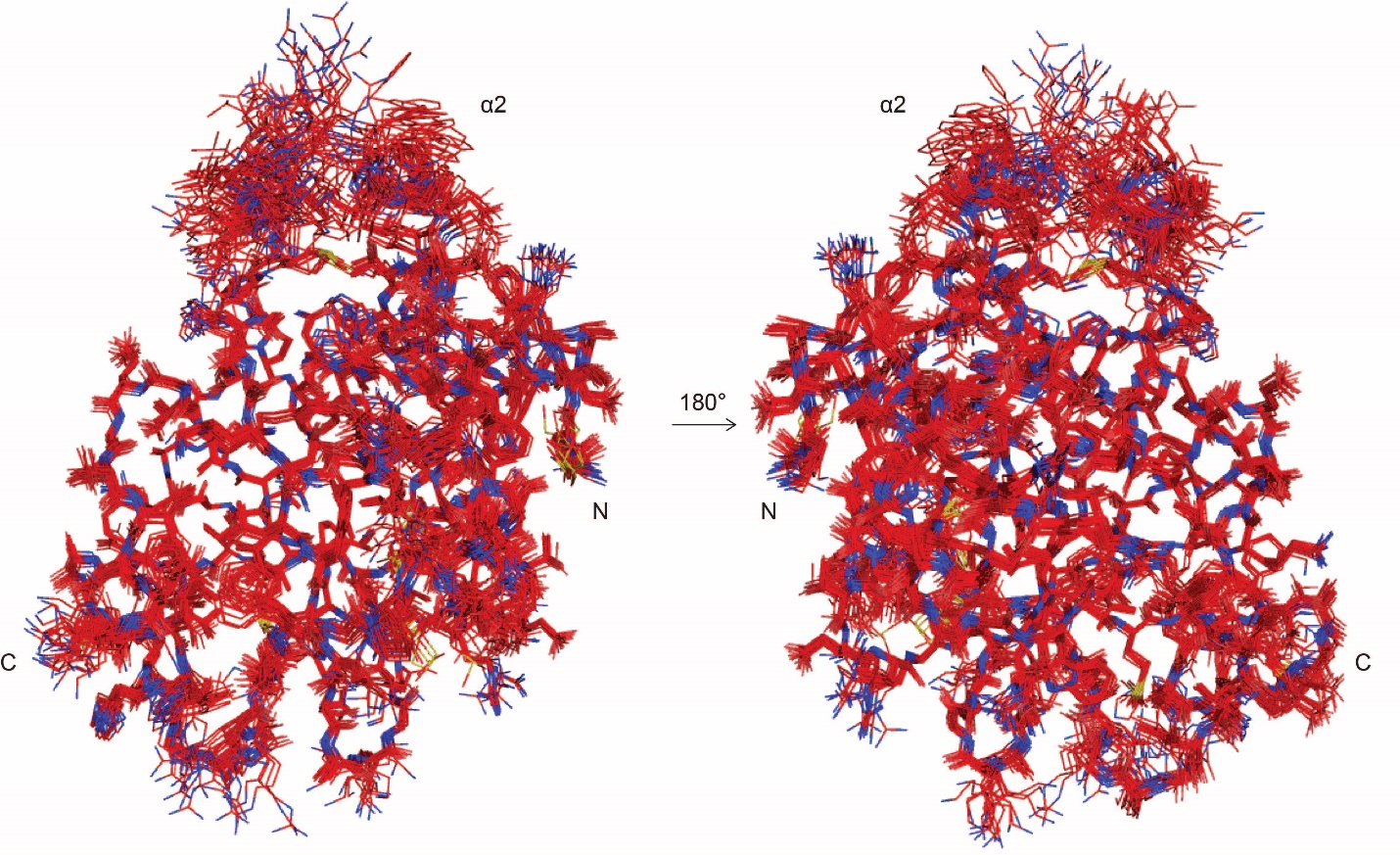


**Supplementary Fig. 4**: NMR ensemble of *Cc* ProXp-ala with all heavy atoms excluding oxygen depicted as lines. Carbon is shown in red, nitrogen in blue, and sulfur in yellow.


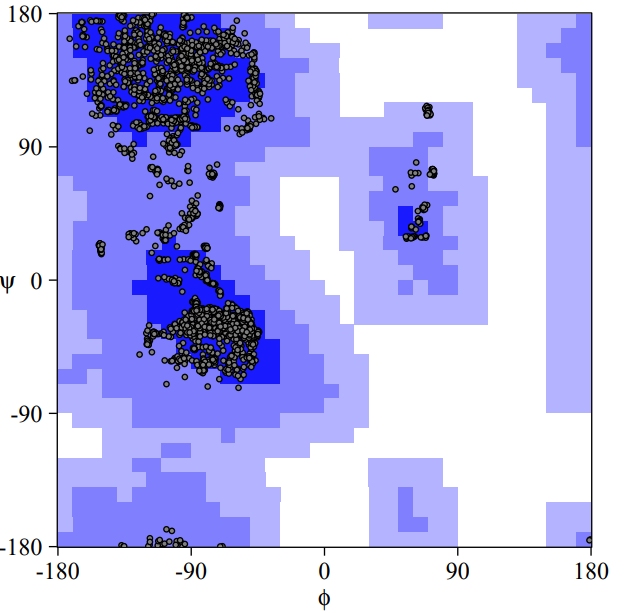


**Supplementary Fig. 5**: Ramachandran plot of *Cc* ProXp-ala NMR ensemble generated by CYANA. 81.2% of Φ,Ψ angles are in most favored regions; 17.5% are in additionally allowed regions; 1.4% are in generously allowed regions; 0.0% are in disallowed regions.
